# Knee osteoarthritis patients assessed during walking for ankle inversion movement discrimination sensitivity

**DOI:** 10.3389/fbioe.2024.1372679

**Published:** 2024-04-18

**Authors:** Yanfeng Huang, Wanjuan Li, Xiaojian Shi, Wenchao Wang, Chengshuo Xu, Roger David Adams, Jie Lyu, Jia Han, Yaohua He

**Affiliations:** ^1^ Department of Orthopedics, Jinshan District Central Hospital Affiliated to Shanghai University of Medicine and Health Sciences, Shanghai, China; ^2^ School of Health Science, Swinburne University of Technology, Hawthorn, VIC, Australia; ^3^ College of Rehabilitation Sciences, Shanghai University of Medicine and Health Sciences, Shanghai, China; ^4^ Research Institute for Sport and Exercise, University of Canberra, Canberra, ACT, Australia; ^5^ Department of Orthopedic Surgery, Shanghai Sixth People’s Hospital Affiliated to Shanghai Jiao Tong University School of Medicine, Shanghai, China

**Keywords:** ambulation, movement discrimination, neuromuscular control, fall risks, global effects

## Abstract

**Background:** Knee osteoarthritis (KOA) is a common musculoskeletal condition that affects dynamic balance control and increases the risk of falling during walking. However, the mechanisms underlying this are still unclear. Diminished ankle proprioception during walking has been found to be related to fear of falling in older adults, with a gender difference in incidence of falling. This study aimed to determine 1) whether ankle inversion proprioceptive acuity during walking is impaired in patients with KOA; and 2) whether there is any difference between genders.

**Methods:** Thirty-two patients with KOA (F:M = 17:15, Median age = 52.5, BMI = 22.3 ± 3.0) and 34 healthy controls without KOA (HC) (F:M = 17:17; median age = 49.0, BMI = 22.5 ± 2.7) were recruited. In patients with KOA, ankle inversion proprioceptive acuity was measured on the affected side using the ankle inversion discrimination apparatus for walking (AIDAW), whilst HC were assessed on a randomly selected side. Two-way (2*2) analysis of variance (ANOVA) was performed to determine the main effects and interaction between gender and KOA condition.

**Results:** Two-way ANOVA showed a significant KOA main effect (F = 26.6, *p* < 0.001, ƞ_p_^2^ = 0.3) whereby AIDAW scores during walking for individuals with KOA were significantly lower than those without KOA (KOA vs. HC: 0.746 ± 0.057 vs. 0.807 ± 0.035). There was neither a gender main effect nor interaction (both *p > 0.05*).

**Conclusion:** Individuals with KOA demonstrated lower ankle proprioception scores during walking compared to their healthy counterparts, with a similar level of impairment in ankle proprioceptive acuity between male and female patients. A low score may contribute to an increased risk of falling in the KOA population. The current findings suggest the need for global concern about lower limb proprioception in the clinical management of KOA.

## Introduction

Knee osteoarthritis (KOA) is one of the most common chronic musculoskeletal diseases affecting mid-aged to older adults. Research has shown that approximately 25% of people over 50 years old worldwide suffer from KOA to varying degrees, with more females than males, and the incidence rate increases significantly with age (Li et al., 2020). In the United States, the annual direct or indirect social and medical expenses caused by KOA are as high as US$128 billion (Litwic et al., 2013). In the long term, the resulting social burden and healthcare expense can be overwhelming (Cross et al., 2014).

Patients with KOA usually complain of pain and stiffness in the knee, and muscle weakness. These conditions can limit physical activities, such as walking. One serious issue associated with KOA during walking is increased risk of falling (Manlapaz et al., 2019). Although some neuromuscular features, such as pain and muscle weakness, have been identified as risk factors of falling for patients with KOA (Cunha et al., 2019; Tayfur et al., 2021), the neuromuscular mechanisms underlying heightened risk of falling are still unclear. One of the important factors in neuromuscular control is proprioception, which has been extensively investigated in patients with KOA. However, there are some issues that are unresolved.

First, most of the studies have focused on proprioception at the knee only, so little is known about the proprioceptive acuity of other lower limb joints, such as the ankle, or about their role during normal function (Salamanna et al., 2023). Although it seems logical to investigate the capability of the “affected” joint, i.e., the knee, to achieve successful movement control in normal function, the brain has to integrate proprioceptive information from multiple joints in the kinetic chain (Han et al., 2016a). During daily and sport activities, the three major lower limb joints, i.e., the ankle, knee and hip, work in concert to maintain dynamic postural control. The ankle is arguably the most important joint, as it is the last joint in the kinetic linkage allowing the lower limbs to interact with the ground (Han et al., 2015). A recent systematic review (Tait et al., 2022) has shown that dynamic ankle measures can precisely estimate peak moments and initial contact angles of the knee. Given the association between ankle proprioception and lower limb neuromuscular control, we assumed that the impaired neuromuscular control as identified in individuals with KOA would be also related to ankle proprioception deficit. However, two studies (Shanahan et al., 2015; Mani et al., 2020) that assessed ankle joint position sense failed to identify any joint position-related proprioceptive impairment in patients with KOA, although this might be due to the fact that the ankle proprioception measured in a non-weightbearing posture did not reflect its role in normal weightbearing functions such as walking (Stillman and McMeeken, 2001). Han et al. (2016) argued that proprioceptive assessment should be ecologically valid and tested in normal functional circumstances, because proprioceptive impairment associated with different chronic musculoskeletal conditions may be task-specific (Han et al., 2022). In accordance with this argument, Shao and others (Shao et al., 2022a) developed an apparatus for assessing ankle proprioception during walking and found that ankle proprioceptive acuity measured in this way was significantly related to fear of falling in older people. Moreover, individuals with chronic non-communicable diseases (e.g., Type 2 diabetes mellitus) showed a high risk of falling (Tilling et al., 2006; Cho and Kim, 2021; Maras et al., 2021), and ankle proprioception deficit was recognized as the strongest predictor on balance impairments in those with chronic stroke (Cho and Kim, 2021). Although patients with KOA were also identified with an increased risk of falling during walking (Wilfong et al., 2023), ankle proprioception has never been assessed during walking in this population.

Second, knowledge about the gender effect often observed in KOA cohorts can be important in relation to gender-specific management in healthcare (Stenberg et al., 2022). This has not previously been considered regarding differences in proprioception between female and male patients with KOA. In general, male adults have been considered to be stronger in the lower limbs than their female counterparts (Ditroilo et al., 2010), and in the KOA cohort, female patients have been reported to have more severe impairments in lower extremity neuromuscular control than male patients. However, a recent large-sample, prospective investigation based in an Asian community suggested that muscle strength was not associated with fear of falling (Mat et al., 2020), signifying the need of further investigations regarding the characteristics of neuromuscular control during walking, as this remains the most common functional activity where fall accidents occur (Promsri et al., 2023). To date, a gender difference on lower limb proprioception has been investigated in healthy cohorts, and some clinical populations (Karkousha, 2016; Lu et al., 2022; Shi et al., 2023a), yet not in KOA (Salamanna et al., 2023). It remains unclear whether ankle proprioception during walking could be affected to different degrees in female and male patients with KOA.

This study aimed to measure ankle proprioception acuity during walking by using a customized ankle inversion discrimination apparatus for testing proprioceptive acuity in walking (AIDAW); make comparisons between individuals with and without KOA; and investigate any gender differences in this cohort. Based on previous findings (van der Esch et al., 2007; Metcalfe et al., 2012; Shao et al., 2022a), we hypothesized that individuals with KOA would show inferior ankle proprioception acuity compared to those without KOA, and that female patients would show worse ankle proprioception compared to their male counterparts.

## Methods and procedure

### Participants

Participants were recruited from Shanghai University of Medicine and Health Sciences and its affiliated hospitals. For the KOA group, the eligible participants were aged over 18 and diagnosed with knee osteoarthritis (KOA), as per the American College of Rheumatology (Altman et al., 1986). For the healthy control group (HC), they were generally healthy and without KOA. The exclusion criteria for both groups included (Li et al., 2020) lower extremity or spine surgery history; (Litwic et al., 2013); ankle injury in the past 6 months or ankle OA; (Cross et al., 2014); visual or vestibular disorders; (Manlapaz et al., 2019); peripheral or central neurological disorders, e.g., stroke; (Cunha et al., 2019); ongoing interventions that could affect the test results, including but not limited to medical, physical therapy, or other alternative treatment. Sample size was determined using G*power software, taking the results from previous work into consideration, where medium to large effect sizes were achieved in comparing ankle proprioceptive acuity between individuals with and without pathological status (Shao et al., 2023a). Finally, 32 individuals with KOA (median age = 52.7, BMI = 22.28 ± 3.00), and 34 healthy controls (HC) (median age = 47.0, BMI = 22.50 ± 2.72) were recruited (Table 1).

**TABLE 1:** Demographic information of participants with and without KOA.

Group		KOA	HC	Statistics
N(F:M)		32 (17:15)	34 (17:17)	X^2^ = 0.139, *p* = 0.71
Age^a^		52.5 (43.0, 56.8)	49 (26.5.0, 67.3)	Z = 0.597, *p* = 0.55
BMI^b^		22.28 ± 3.00	22.50 ± 2.72	t = 0.318, *p* = 0.75
KOOS^b^	Pain	71.61 ± 8.82		
	symptom	59.23 ± 11.07		
	Daily living	80.57 ± 8.34		
	Sport	54.41 ± 17.22		
	QoL	56.67 ± 15.17		

^a^ Median (Q1, Q3).

^b^ Mean ± SD.

^c^ Statistical significance.

ES, effect size; HC, health control; BMI, body weight/height^2; KOA, knee osteoarthritis; KOOS, the knee injury and osteoarthritis outcome score.

The symptom severity resulting from KOA was measured by the knee injury and osteoarthritis outcome scale (KOOS), which has five subscales regarding pain and other symptoms, activities of daily living, sports and recreational activities, and quality of life, respectively. Each subscale can score individuals with KOA from 0 to 100, with lower scores representing more severe knee conditions. Compared to another commonly-used clinical instrument, the Western Ontario and McMaster University Osteoarthritis Index (WOMAC) (Baron et al., 2007), KOOS is more suitable for relatively young and active populations with a problematic knee (Roos and Lohmander, 2003). In this study, we used a validated Chinese version of the KOOS, with all subscales having good to excellent reliability (ICC = 0.80–0.97) (Yang et al., 2023), Participants were expected to complete this questionnaire in 10 min.

### Proprioceptive assessment

Ankle proprioceptive assessment was performed on the affected side for individuals with unilateral KOA or the worse side of the bilateral KOA cohort was selected to assess to avoid additional testing of bilateral KOA participants, and on a randomly selected side for the HC group. Ankle proprioceptive acuity was assessed using the ankle inversion discrimination apparatus for walking (AIDAW). The AIDAW is one variant of ecologically valid proprioception testing systems that use signal detection principle-based analysis of ankle inversion discrimination data. The AIDAW was modified to incorporate an ambulation function into ankle proprioception assessment to achieve high ecological validity. In previous work, the AIDAW has been identified as a reliable and valid instrument that can be used to detect ankle proprioception deficits resulting from musculoskeletal dysfunction and aging (Shao et al., 2022a; Shao et al., 2023b).

**FIGURE 1:**
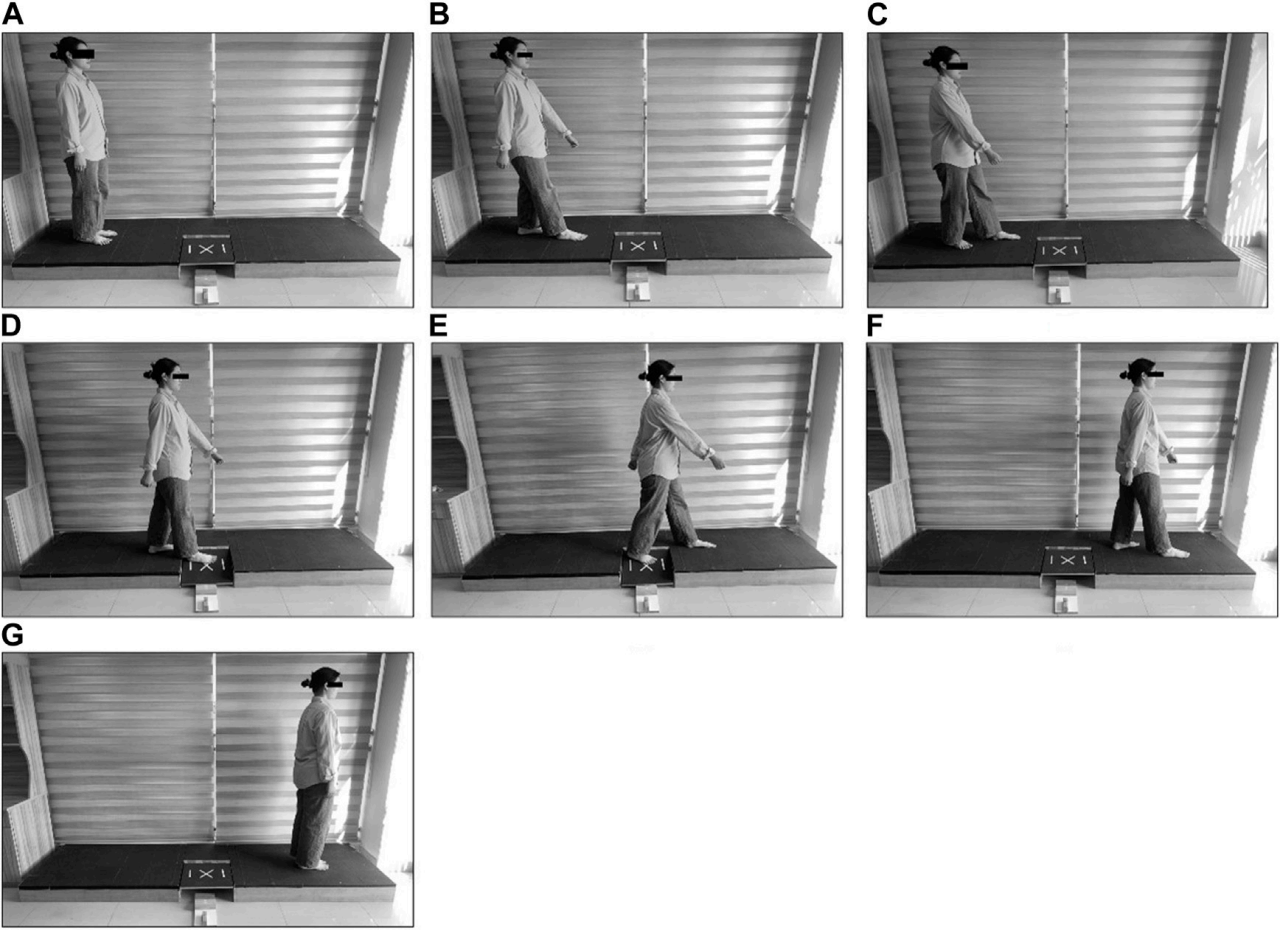
Ankle inversion discrimination assessment for walking With the testing procedure **(A–G)**, from the starting to the sixth steps for the right side and the testing scene for right side.

The AIDAW consists of a walking platform (285 cm*80 cm*16 cm) and a movable board (45 cm*42 cm*1.2 cm) hinged on one side of the middle section. Under the movable board is a set of springs that are used to offer elastic support and a set of physical stops made of four wooden blocks that are used to terminate the movement of the board at four different tilts in the sagittal plane. The tilts are from Position 1, with a 10-degree tilt, to Position four of a 16-degree tilt, and a 2-degree increment between each adjacent position. Participants were instructed to stand on one end of the AIDAW in bare feet, with eyes open, looking straight forward, and the testing side in line with the centre of the movable testing board, and then to initiate self-paced gait by the contralateral leg, stepping onto the movable board on the testing side at the third step where they were to perceive the extent of ankle inversion, and then without stopping, continue to walk three steps to the other end of the platform (Figure 1).

The standard testing procedure included a familiarisation part and a formal testing part. In the familiarisation part, individuals are given 12 trials in which they were required to walk on the platform and experience the four different positions, in a sequence from the shallowest to the deepest, 3 times, giving 3 presentations for each position. In the formal testing part, the movement tasks were as before, with the tilt extent of the movable board varied randomly. The participant was asked to make absolute position judgments regarding the extent of ankle inversions by orally reporting the perceived position using the numbers one to four. After each trial, participants could continue to the next trial or request a rest of up to 1 min. In total, 40 trials were completed, with each of the four different tilt position presented 10 time in a random sequence, taking around 20 min for each participant to complete. One investigator was responsible for manipulating the physical stops and recording the reported position judgments. No feedback was provided regarding the actual position during formal testing.

### Statistical analysis

Ankle proprioception scores were generated using a nonparametric analysis derived from signal detection theory (Stanislaw and Todorov, 1999). Specifically, the perceived position judgment, and the actual position were used to generate a receiver operating characteristic curve (ROC). The average area under the curves (AUC) based on the judgments from three pairs of adjacent positions (i.e., 1vs 2, 2v3, 3 v4) was calculated to represent a participant’s overall ankle proprioceptive acuity, where an AUC value equal to 0.5 represented making position judgments by chance, and a value equals to 1.0 represented perfect position judgments. More details regarding the AUC calculation can be found in Supplementary Material.

SPSS 25.0 was used to perform the statistical analysis. The Wilcoxon-Mann-Whitney test and Chi-square test were used to check the between-group comparability of anthropological parameters, i.e., age, gender, body mass index (BMI). Two-way analysis of variance (ANOVA) was used to investigate the main effect and the interaction between gender and KOA, with significance level set at 0.05. Effect size was calculated using partial Eta squared (ƞ_p_^2^), with a ƞ_p_^2^ value equal to 0.01 representing a small effect size, 0.06 a medium effect size, and 0.14 a large effect size, respectively (Miles and Shevlin, 2001). Once a significant interaction is found, *post hoc* analysis via Bonferroni *post hoc* tests would be conducted to identify the sources of the differences.

## Results

**FIGURE 2:**
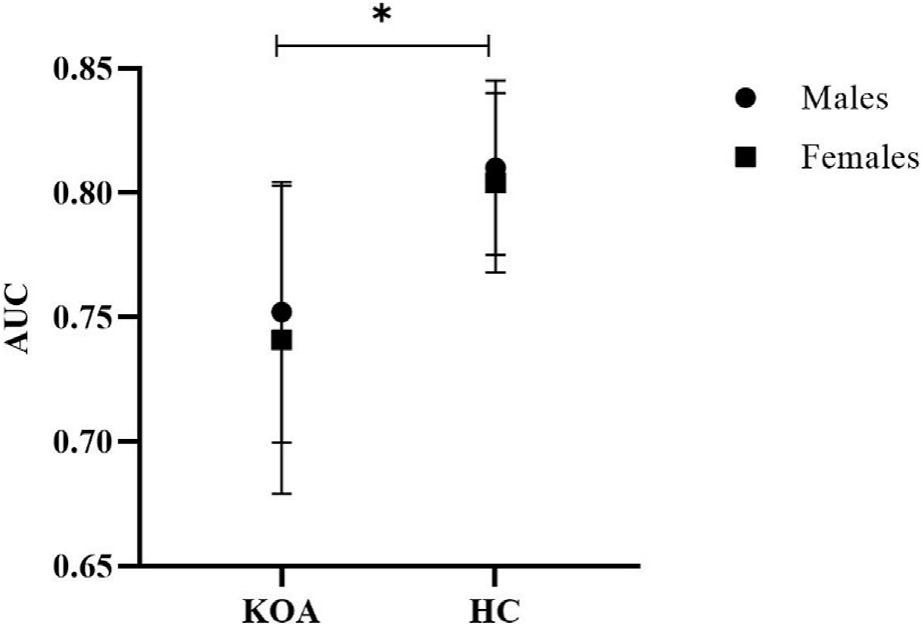
Ankle inversion discrimination apparatus (AIDAW) proprioceptive comparison between KOA and HC, both with males and females *p < 0.05, indicative of statistically significant intergroup difference; the error bars refer to standard deviation associated with mean AUC values.

All anthropological parameters were comparable between KOA and HC groups (all *p* > 0.05). Two-way ANOVA showed a significant KOA main effect (F = 26.6, *p* < 0.001, ƞ_p_^2^ = 0.3) (Figure 2). Ankle inversion movement discrimination sensitivity during walking for individuals with KOA was significantly lower than those without KOA (KOA vs. HC: 0.746 ± 0.057 vs. 0.807 ± 0.035). There was neither a significant gender main effect (F = 0.06, *p* = 0.449, ƞ_p_^2^ = 0.001) nor interaction (F = 0.58, *p* = 0.803, ƞ_p_^2^ = 0.009).

## Discussion

In this first study to investigate ankle proprioception acuity for individuals with KOA during functional walking, individuals with KOA showed impaired ankle proprioceptive acuity compared to those without KOA, whereas there was no significant difference in ankle proprioception scores between male and female participants. The current findings extended the previous observations on knee proprioception and imply that there is a need for global concern about lower extremity neuromuscular control in this cohort.

Methodological heterogeneity has abounded in terms of assessing proprioception in KOA cohorts, which may result in the inconsistent findings across the previous studies (Shanahan et al., 2015; Mani et al., 2020). In Mani et al.’s work (Mani et al., 2020), the joint position reproduction (JPR) and the threshold to detection of passive motion (TTDPM) methods were both used to investigate proprioceptive acuity in the knee and the adjacent joints, comparing individuals with and without early-stage KOA, and the results showed that joint position sense in the lower limb joints was comparable, whilst ankle movement sense showed significant inter-group differences. These findings suggest that previous joint position sense assessment research may be not specific enough to detect proprioceptive impairment in this population. Han et al. (Han et al., 2016b) argue that during functional movement, the brain has to use both position and movement information to determine body movement status in 3D space. In line with this argument, the active movement extent discrimination assessment (AMEDA) has been designed to consider that the actual working circumstances of proprioception, that involve using information from both position and movement senses, and the system can assess proprioception during functional movement, such as walking and landing (Han et al., 2021; Shi et al., 2023b). Specifically, in our previous work, the AMEDA apparatus involving walking, i.e., the AIDAW, showed scores with a significant negative correlation with fear of falling. That is, high proprioceptive acuity was associated with low fear of falling, and *vice versa*. In the current work, results showed that the AIDAW test was able to detect ankle proprioception impairment in patients with KOA. Furthermore, the current study showed a large effect size for the proprioceptive acuity difference between individuals with and without KOA, when the test involved incorporating walking into the proprioception assessment, compared to small to medium effect sizes obtained by using the other methods (Shanahan et al., 2015; Mani et al., 2020). Another empirical comparison between JPR and AMEDA was carried out by Steinberg and others to investigate discriminative ability to identify individuals with and without recent ankle injuries. In this work, injury effect was demonstrated only using AMEDA method, which was further amplified when this assessment was performed under weight-bearing condition (Steinberg et al., 2019). Together, these observations suggested that ankle proprioception assessment merely targeting position sense may not be specific enough to detect proprioceptive impairments in this population. In contrast, a function-integrated proprioception testing protocol, i.e., AIDAW, under weight-bearing condition may be more specific for detecting proprioceptive impairment in the ankle joint adjacent to the knee with KOA, consistent with the ecological validity argument about proprioceptive assessment methods (Han et al., 2016b).

Although previous studies have found no significant correlation in proprioception scores between the ankle and knee joins (Han et al., 2013a; Han et al., 2013b), the ankle proprioception impairment detected in the KOA cohort may be ascribed to a central mechanism resulting from KOA. Research has suggested that KOA may cause loss of mechanoreceptors, e.g., Golgi corpuscles, Ruffini corpuscles, and fusimotor hyperactivity abnormally induced by nociceptive stimulation, which contribute to peripheral impairments on joint position sense and movement sense in the affected knee joint (Salamanna et al., 2023). No recent ankle injury history or ankle OA existed in the recruited KOA cohort in this study, indicating a possible altered central processing on neuromuscular control in the lower limb may exist in the KOA cohort. A recent systematic review suggested that individuals with KOA showed a series of structural and functional brain changes (Salazar-Méndez et al., 2023). Notably, in this clinical cohort, the reduction in grey matter was detected in the primary somatosensory cortex and the decreased connectivity bilaterally in the parietal lobe. A similar phenomenon was also shown in other musculoskeletal disorders, such as non-specific chronic low back pain, with a global impairment in lower limb proprioception (Xiao et al., 2022). Together, the current findings indicate that the presence of KOA may have a generalized effect on lower limb neuromuscular control., through a central mechanism, which may be a potential factor contributing to increased risk of falling. Again, this points to the need for a global concern regarding lower limb neuromuscular control in the management of KOA.

This study was also powered to explore possible gender differences in the KOA cohort in ankle proprioceptive acuity. Recently, gender-specific healthcare management has received increasing concern. Knowledge regarding any gender differences in KOA cohorts can be important for informing gender-specific management in healthcare (Stenberg et al., 2022). For instance, in the contemporary view of graded intervention in KOA, exercise-based programs have been recognized as the primary treatment in international guidelines (Teo et al., 2019; Jackie et al., 2022). To enhance the therapeutic impact, compliance, and the cost-effectiveness of healthcare management, it is yet to be determined whether exercise-based conservative programs should incorporate more proprioceptive training or muscular strengthening, and *vice versa*, or it may not matter equally for both genders. Previous work has suggested females tend to be more vulnerable in terms of neuromuscular dysfunction resulting from KOA (Labanca et al., 2021). Nevertheless, an early work from Van der Esch and others (van der Esch et al., 2007) suggested that knee joint proprioceptive deficits in individuals with KOA were not confounded by gender differences. In accordance with this finding, the current work has extended this notion and suggested that regardless of KOA conditions, males and females showed no significant difference in ankle proprioceptive acuity during walking. Therefore, clinical practitioners should prescribe similar treatment protocols to male and female patients with KOA.

There were some strengths and limitations regarding this study. Firstly, the current study provided a cost-effective and user-friendly ankle proprioceptive assessment tool that can be employed in KOA clinics. Secondly, in comparison to the other instrumental devices, the AIDAW is sufficiently sensitive and of high enough external validity to detect lower extremity proprioceptive impairments resulting from KOA. However, the affected limb or the worse side of the KOA cohort was primarily selected to be assessed and compared, without distinguishing unilaterality or bilaterality of KOA, which might overestimate ankle proprioception performance, when it comes to a wider KOA cohort, given high prevalence of bilateral KOA in real life (Metcalfe et al., 2012). Then, the recruited age range (i.e., aged from 43 to 56.8 years) in this present study tended to be relatively younger than those reported in previous studies, which may undermine the comparability between the current findings and those from previous reports. Notably, it has been reported that there is an increasing number of relatively young people diagnosed with KOA (Ackerman et al., 2017), therefore the current work may help to understand the lower limb proprioception impairments in KOA across the lifespan. The current results suggest even in a relatively younger cohort with KOA, ankle proprioception impairments were evident, which implicates intervention in the early stage. More work involving a large sample to investigate the interaction between aging and proprioception is expected in the KOA population.

## Conclusion

Ankle proprioceptive acuity was impaired in patients with KOA, regardless of gender, and this fact may contribute to an increased risk of falling in this population. The current findings suggest the need for global concern about lower limb proprioception in the clinical management of KOA, and ankle proprioceptive acuity during walking should be assessed and managed in clinical practice.

## Data Availability

The original contributions presented in the study are included in the article/Supplementary Material, further inquiries can be directed to the corresponding authors.
