# Supplementary material for: Knee osteoarthritis patients assessed during walking for ankle inversion movement discrimination sensitivity
Source: Front Bioeng Biotechnol. 2024 Apr 18;12:1372679. doi: 10.3389/fbioe.2024.1372679 (PMC11063258; doi:10.3389/fbioe.2024.1372679)
Supplement: Supplementary file 1 [file Presentation1.pdf]

Supplement 1: Data analysis procedure

Active inversion extent discrimination assessment for walking (AIDAW) was used to quantify proprioception acuity. In this method, Individuals are required to make absolute position judgments on three paired adjacent positions, i.e. 2v1,3v2 4v3. As per signal detection theory, the receiver operating characteristics (ROC) curve was drawn based on the position-response matrix, and the area under the curve (AUC) was calculated to present the discrimination scores. The AUC has upper (1.0) and lower (0.0) boundaries where 1.0 represents perfect proprioceptive acuity during walking, 0.5 is chance responding, and 0.0 means inability to judge the differences between positions correctly. This statistical method has been widely used in perception-related research work(Han, 2013; Macmillan & Creelman, 2004; Stanislaw & Todorov, 1999). For a better understanding of this statistical procedure, in this supplement document, we present a step-by-step procedure regarding how to generate discrimination scores, seen as the six steps in the following:

STEP 1

A raw data sheet recording the 40 position-response pairs were retrieved from a sampled participant (Table S1.1), once the completion of a formal testing part of AIDAW.

Table S1.1 An example of raw data sheet

Trial	Actual Position presented	Participant's Response
1	2	2
2	4	3
3	1	2
4	1	1
…	…	…
…	…	…
36	3	2
37	4	5
38	2	2
39	4	4
40	3	3

Forty trials were conducted in one assessment session. The second column (Position) represents the randomized sequence of the four pre-set position stimuli, with number 1 representing the shallowest ankle inversion position, and so on, number 4, the deepest position. The third column (Response) represent the response from a sampled participant.

STEP 2

The overall proprioception performance for a sampled participant was quantified based on the mean discrimination scores from the three comparisons between adjacent positions, i.e.2v1, 3v2 and 4v3. To achieve that, data from table S1 was used to convert a table for the frequency of response at each position stimuli (Table S1.2).

Table S1.2 Amounts of responses for the four positions in AIDAW

	**Response**			
**Position**	1	2	3	4
1	5	4	1	0
2	1	5	3	1
3	1	4	4	1
4	0	0	3	7

STEP 3

Table S1.2 was further converted and then divided to three cumulative frequency tables (Table S1.3,1-3).

Table S1.3.1 Cumulative frequency of response for position 1 and position 2 in AIDAW

	Response			
Position	1	1 and 2	1,2 and 3	1,2,3, and 4
1	5	9	10	10
2	1	6	9	10

Table S1.3.2 Cumulative frequency of response for position 2 and position 3 in AIDAW

	Response			
Position	1	1 and 2	1,2 and 3	1,2,3, and 4
2	1	6	9	10
3	1	5	9	10

Table S1.3.3 Cumulative frequency of response for position 3 and position 4 in AIDAW

	Response			
Position	1	1 and 2	1,2 and 3	1,2,3, and 4
3	1	5	9	10
4	0	0	3	10

STEP 4

The three cumulative frequency tables were further converted to three tables regarding cumulative

probabilities for each position comparison. (Table S1,4,1-3), by using the cumulative frequency being divided by the total number of trials for each position, i.e. 10.

Table S1.4.1 Cumulative probabilities of response for position 1 and position 2 in AIDAW

	Response			
Position	1	1 and 2	1,2 and 3	1,2,3, and 4
1	0.5	0.9	1.0	1.0
2	0.1	0.6	0.9	1.0

Table S1.4.2 Cumulative probabilities of response for position 2 and position 3 in AIDAW

	Response			
Position	1	1 and 2	1,2 and 3	1,2,3, and 4
2	0.1	0.6	0.9	1.0
3	0.1	0.5	0.9	1.0

Table S1.4.3 Cumulative probabilities of response for position 3 and position 4 in AIDAW

	Response			
Position	1	1 and 2	1,2 and 3	1,2,3, and 4
3	0.1	0.5	0.9	1.0
4	0	0	0.3	1.0

STEP 5

Excel 2016 was performed to draw the receiver operating characteristic (ROC) curve based on data from table S1.4.1-3, seen as one example for the position 1 and position 2 discrimination (Figure S1).

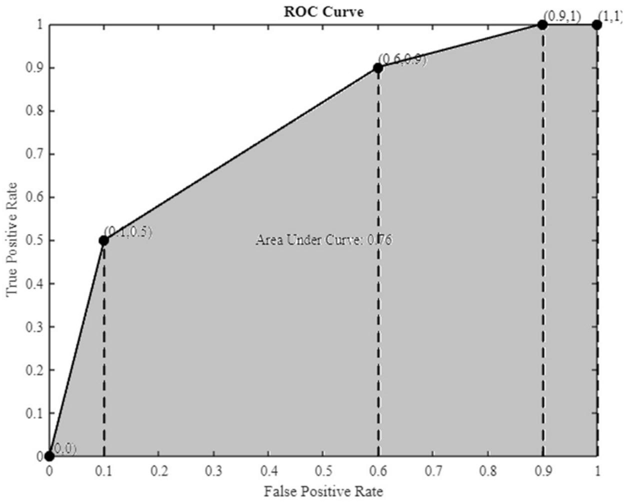

Figure S1 The Receiver Operating Characteristic (ROC) Curve for discrimination ability between

Position 1 and 2 in a participant. The shaded area represents the area under the ROC curve (AUC).

STEP 6

The area under the ROC curve (AUC) for each position comparison (i.e. 2v1,3v2 and 4v3) can be retrieved via the geometry area formulas (Figure S1). In this case, the AUC values for 2v1 position discrimination were 0.76(the shaded area), and accordingly, the same procedure can be used to retrieve the other AUC values for 3v2 and 4v3, based on data from Table 1.4.2-3, which were 0.540 and 0.875 respectively. Overall, the mean AUC for three pairs was 0.725 (see the formula below), indicating that this sampled participant's ability to discriminate between four preset ankle inversions during walking was better than random guessing (0.5), but not yet perfect (1.0).

$$Mean\ AUC = (AUC\ 2v1 + AUC\ 3v2 + AUC\ 4v3)/3$$

References

Han, J. (2013). *Multiple joint proprioception in movement discrimination*. University of Canberra.

Macmillan, N. A., & Creelman, C. D. (2004). *Detection theory: A user's guide*. Psychology press.

Stanislaw, H., & Todorov, N. (1999). Calculation of signal detection theory measures. *Behavior Research Methods, Instruments, & Computers*, *31*(1), 137-149. https://doi.org/10.3758/BF03207704
